# Editorial: Bilingual and Multilingual Spoken-Word Recognition: Empirical and Theoretical Perspectives

**DOI:** 10.3389/fpsyg.2021.696354

**Published:** 2021-06-02

**Authors:** Michael C. W. Yip, Henrike K. Blumenfeld, Anna B. Cieślicka

**Affiliations:** ^1^Department of Psychology, The Education University of Hong Kong, Hong Kong, China; ^2^School of Speech, Language, and Hearing Sciences, Bilingualism and Cognition Lab, San Diego State University, San Diego, CA, United States; ^3^Department of Psychology and Communication, Brain and Cognition Lab, Texas A&M International University, Laredo, TX, United States

**Keywords:** bilingual spoken-word recognition, influencing factors on spoken word processing, multilingual, top-down information, bottom-up information

Spoken-word recognition is a fundamental aspect of language comprehension. Over the last four decades, a great number of studies have laid a solid foundation on spoken word recognition in monolinguals (e.g., Marslen-Wilson, 1987, McClelland and Elman, 1986; Luce and Pisoni, 1998). With bilingualism and multilingualism being the world-wide norm rather than the exception (Grosjean, 2014), strong foundations have also been laid on spoken-word comprehension in bilinguals, rooted in classical frameworks from monolinguals and formalized in models such as the Bilingual Model of Lexical Access (BIMOLA, Grosjean, 1997; Léwy and Grosjean, 2008) and the Bilingual Language Interaction Network for Comprehension of Speech (BLINCS, Shook and Marian, 2013). Core features of these models include crosslinguistic interactive activation of representations, language non-selective bottom-up activation of auditory input, and top-down modulation of activation to guide lexical selection across two languages. The bottom-up word recognition process, albeit non-selective, is known to be influenced by the nature of phonological categories of L2 learners (e.g., Broersma and Cutler, 2011) and by the presence of acoustic features of the non-target language in auditory input (Ju and Luce, 2004). Finally, self-organizing features of recent computational models allow us insight into the dynamic emergence of the bilingual lexicon during learning and with continued experience (Li and Farkas, 2002; Shook and Marian, 2013). Broadly, Grosjean's (1989) often-cited statement continues to ring true: “bilinguals are not two monolinguals in one.” The current Research Topic contributes to the effort to further specify spoken word recognition in bilingual populations by contributing new data across ages, bilingual populations, and processing levels. Themes that emerged across the four contributed articles include the nature of mental representations and processes involved in early bilinguals' spoken-word recognition, and bottom up perceptual processing in late bilingual low-proficient L2 listeners.

De Anda and Friend and Akhavan et al. extend current knowledge on the emergence and nature of bilingual cognitive architectures in young children and college-aged adults, respectively. De Anda and Friend show that cross-linguistic interaction at the lexical level is evident even in very young emerging bilinguals: 18 and 24 month old toddlers who are exposed to both Spanish and English at least 20% of the time per language. To explore the development of lexical-semantic associations in bilingual toddlers, the authors employed a computerized comprehension test (CCT), a behavioral measure capturing children's ability to touch the image on the screen in response to a verbal sentence prompt. In addition, the Intermodal Preferential Looking Task was used to investigate lexical-semantic priming. Lexical semantic priming was examined longitudinally in Spanish-English bilingual toddlers aged 18 and 24 months. Of interest was whether within-language vocabulary size, the number of known translation equivalents, or total conceptual vocabulary size, would be significant predictors of bilingual children's lexical-semantic processing. Results revealed that measures of within-language receptive vocabulary were not good predictors of bilingual children's lexical-semantic processing. Instead, the size of children's total conceptual vocabulary and number of translation equivalents were both positively correlated with the degree of the demonstrated lexical-semantic priming. This finding confirms an integrated lexicon even in very young simultaneous bilinguals. In a group of adult (mean age = 20) proficient early Spanish-English bilinguals who had learned both languages by age 6 and continued to use Spanish about 26% of the time, Akhavan et al. showed bilingual-monolingual differences in the processing of dominant-language complex syntax. The authors found that the bilinguals showed less interference than monolingual controls between competing noun phrases during auditory comprehension of object-relative sentences. This earlier competition resolution in bilinguals was linked to better performance on a high-conflict N-back task, indexing working memory and inhibitory control. This finding suggests that top-down cognitive processes that modulate competition in the bilingual language system may also be engaged when bilinguals resolve interference in language-specific contexts and at the sentence level.

In contrast to the focus on early bilinguals from typologically similar languages in De Anda and Friend and Akhavan et al.'s contributions, Wiener and Lee and Simeon-Yasufuku and Doyle discuss data from late bilinguals in typologically-different languages. These data focus on bottom-up perceptual processing of single words during challenging listening conditions, an area where late bilinguals are known to struggle in their non-native language (L2, Flege, 1995). Wiener and Lee examined whether English-native adult L2 learners of Mandarin, with an average of 3.6 years of classroom exposure, would focus more on knowledge (probability information)-based strategies over acoustic (speaker normalization)-based processes during spoken word identification in multi-talker speech. The authors found that, by a gating experiment, these Mandarin-L2 listeners relied heavily on a top-down knowledge-based approach during word identification. Instead, the control group of native Mandarin speakers switched to acoustic strategies earlier than the Mandarin-L2 listeners as more input became available during the spoken word processing. The authors argue that these findings, which were evident in multi-talker over single-talker contexts, could be linked to the limited experience of L2 speakers with speaker normalization in Mandarin. Similarly, Simeon-Yasufuku and Doyle examined how young adult native speakers of Japanese who had studied English for at least 3 years in Japan and were now attending an American university identified spoken non-words. More specifically, Simeon-Yasufuku and Doyle explored the Phonological-Superiority Hypothesis and the Phonetic-Superiority Hypothesis, both of which address the issue of how phonological representations are acquired in sequential multilinguals. While the former places emphasis on differing L1 syllabification preferences as a source of cross-linguistic influence in L2 speech perception, the latter capitalizes on acoustic characteristics of speech sounds which make their perception challenging when placed in some word positions. Participants transcribed non-words based on either auditory or auditory-visual input in a McGurk setup where audio and visual information was at times inconsistent. Critically, the non-words contained stop consonants in syllable coda positions that are legal in English but heavily restricted in Japanese. Thus, the authors could test to what extent the Japanese-English late bilinguals would perform based on L1 or L2 biases. Performance could not be explained solely on the basis of acoustic informativity or phonological biases of participants' L1 and hence no support was found for either the Phonological- or the Phonetic-Superiority Hypothesis. Instead, findings suggest that the Japanese-English participants, who listened to unfamiliar words in a multi-talker babble context, paid attention to and integrated multiple cues, including acoustic, visual, and phonological information. The authors thus propose a cognitive cue integration framework to account for the L1 phonological influences on speech perception in the second/foreign language.

Taken together, De Anda and Friend and Akhavan et al. show that early emerging bilinguals already possess a fundamentally integrated and interactive crosslinguistic lexicon; and that, in young adulthood, the lifelong experience of bilingualism may yield differences relative to monolingualism in how interference is resolved during language-specific sentence-level processing. Wiener and Lee and Simeon-Yasufuku and Doyle show that, in challenging listening environments, low-proficient late bilinguals supplement bottom-up acoustic input in their L2 with other, top-down, information, including previous lexical knowledge and visual cues that are available in the environment. We hope that the current Research Topic will stimulate further research into the emergence and development of cognitive architectures that support bilingual comprehension, and into the specifics of how top-down processes are engaged to support challenging auditory bottom-up processing.

## Author Contributions

All authors listed have made a substantial, direct and intellectual contribution to the work, and approved it for publication.

## Conflict of Interest

The authors declare that the research was conducted in the absence of any commercial or financial relationships that could be construed as a potential conflict of interest.
